# Fabrication and Advanced Imaging Characterization of Magnetic Aerogel-Based Thin Films for Water Decontamination

**DOI:** 10.3390/gels10060394

**Published:** 2024-06-11

**Authors:** Adelina-Gabriela Niculescu, Bogdan Mihaiescu, Alexandra Cătălina Bîrcă, Alina Moroșan, Oana Maria Munteanu (Mihaiescu), Bogdan Ștefan Vasile, Tony Hadibarata, Daniela Istrati, Dan Eduard Mihaiescu, Alexandru Mihai Grumezescu

**Affiliations:** 1 Department of Science and Engineering of Oxide Materials and Nanomaterials, National University of Science and Technology Politehnica Bucharest, 011061 Bucharest, Romania; adelina.niculescu@upb.ro (A.-G.N.); bogdan.mihaiescu@upb.ro (B.M.); alexandra.birca@upb.ro (A.C.B.); oanamihro@yahoo.co.uk (O.M.M.); bogdan.vasile@upb.ro (B.Ș.V.); tony.hadibarata@upb.ro (T.H.); grumezescu@yahoo.com (A.M.G.); 2 Research Institute of the University of Bucharest—ICUB, University of Bucharest, 050657 Bucharest, Romania; 3 Department of Organic Chemistry, National University of Science and Technology Politehnica Bucharest, 011061 Bucharest, Romania; alina.morosan@upb.ro (A.M.); daniela.istrati@upb.ro (D.I.); 4 Department of Environmental Engineering, Faculty of Engineering and Science, Curtin University Malaysia, CDT 250, Miri 98009, Malaysia

**Keywords:** aerogel-based thin films, magnetic silica-based aerogels, advanced imaging techniques, infrared microscopy, MALDI, water decontamination

## Abstract

Aerogels have emerged as appealing materials for various applications due to their unique features, such as low density, high porosity, high surface area, and low thermal conductivity. Aiming to bring the advantages of these materials to the environmental field, this study focuses on synthesizing magnetic silica aerogel-based films suitable for water decontamination. In this respect, a novel microfluidic platform was created to obtain core-shell iron oxide nanoparticles that were further incorporated into gel-forming precursor solutions. Afterward, dip-coating deposition was utilized to create thin layers of silica-based gels, which were further processed by 15-hour gelation time, solvent transfer, and further CO_2_ desiccation. A series of physicochemical analyses (XRD, HR-MS FT-ICR, FT-IR, TEM, SEM, and EDS) were performed to characterize the final films and intermediate products. The proposed advanced imaging experimental model for film homogeneity and adsorption characteristics confirmed uniform aerogel film deposition, nanostructured surface, and ability to remove pesticides from contaminated water samples. Based on thorough investigations, it was concluded that the fabricated magnetic aerogel-based thin films are promising candidates for water decontamination and novel solid-phase extraction sample preparation.

## 1. Introduction

Aerogels are mesoporous gels that have a gas as the dispersing phase instead of a liquid. Aerogels are appealing due to their unique properties, including low density, high porosity, high surface area, low thermal conductivity, good optical transparency, and superhydrophilicity [1,2]. Aerogels have opened a new field of research and breakthroughs thanks to their features, and more inexpensive and sustainable synthesis methods are bringing them closer to large-scale production [3]. The main route of obtaining aerogel structures is the sol-gel process, which allows the formation of uniform porous materials while enabling flexibility around reagents, reaction conditions, and further processing steps [4,5,6,7].

There are different approaches to the methods of film formation, from common, conventional methods like dip coating, spin coating, spray methods, chemical vapor deposition, and tape casting [8] to more advanced methods like 3d printing [9,10], inkjet printing [9], and plasma spraying [11]. Chemical structure is one of the most important factors in determining aerogel coating properties, with silica aerogels being the most widely studied and used. However, more and more new precursors for simple or composite aerogel films have been used in recent years, like biopolymers such as cellulose or chitosan [12,13,14,15], other polymers like polyimide with low water uptake [16] or polyaniline as conductive material [17,18] or carbon-based aerogel composite [12,17,19,20]. Another important aspect of aerogel production is the drying method, which determines the material’s pore structure and whether it is maintained or will suffer degradation. Supercritical dried gels, although via a very expensive method, exhibit pristine pore structure and little degradation compared to freeze-dried or ambient-dried gels. Nonetheless, ambient pressure drying tends to become the standard in aerogel fabrication due to the low cost of production and the recent development of methods for maintaining its microstructure [4,15,21,22].

Given their versatility and promising potential, aerogel-based thin films can be used in a wide range of applications. Aerogel coatings are especially popular in sensing applications, like underwater acoustic transducers made, composed of piezoelectric aerogels from lead zirconate titanate [23], humidity sensors [24], or volatile organic compound selective sensors made from carbon nanotube-based aerogel film [25]. Another great application for aerogel films from polyamide, methyl silsesquioxane, polyurethane, and silica is in composites that serve for integration into circuits like microwave strip lines or low capacitance chip connectors, intermetal dielectric layers, and dry etching or gap filling in different configurations or microelectronics [26,27,28,29,30]. Conductive films can be used as electrodes or supercapacitors [14,18,31,32].

However, there is limited literature on aerogel thin films and coatings for water decontamination; other forms of aerogels, like membranes or monoliths, are a more popular option [33,34,35]. Thus, aiming to fill this research gap and bring the advantages of thin films to the environmental field, this study focuses on utilizing cheap and reliable fabrication methods to obtain magnetic silica aerogel-based films suitable for water decontamination.

To fully leverage the benefits of aerogel films, advanced imaging techniques are crucial for thorough characterization. Therefore, a series of imaging techniques can be employed, such as scanning electron microscopy (SEM), transmission electron microscopy (TEM), atomic force microscopy (AFM), matrix-assisted laser desorption/ionization (MALDI), Fourier transform infrared (FT-IR) microscopy, optical microscopy, and X-ray microtomography. However, each of these techniques presents certain limitations and drawbacks on its own. For instance, using SEM for non-conducting materials implies the deposition of conductive compounds like gold, which can modify the sample surface and conceal actual morphological traits [36]. Moreover, fragile structures like aerogel thin films can degrade under the electron beam, damaging the analyzed sample and leading to misinterpretation of its structure [37]. Similarly, TEM assumes complex sample preparation and provides detailed information on very small sample portions, which may not be representative of the entire film [38,39]. Despite its great resolution, AFM allows the depiction of surface topography with limited depth information and suffers from slow scan speeds for high-resolution imaging [40]. On the other hand, FT-IR microscopy is mainly surface-sensitive, offering limited insights into the bulk properties of materials and lower spatial resolution compared to other imaging methods [41]. The above-mentioned drawbacks highlight the need for more advanced and integrated imaging techniques to provide complete characterization of aerogel-based thin films. Thus, this study proposes the use of combined imaging techniques, completing and correlating generated information from each analysis.

Given the importance of aerogel films and coatings and the scarce literature regarding water decontamination and spectrometer-coupled microscopy characterization of aerogels, our work brings different advanced imaging methods for the characterization of thin films. In more detail, this study comprises the fabrication of magnetic silica aerogel-based thin films, their advanced physicochemical characterization, and qualitative evaluation of their potential application in pesticide adsorption from contaminated water samples (related to both water decontamination and solid-phase extraction for analytical purposes).

## 2. Results and Discussion

The aerogel thin films were successfully obtained via dip coating, proving high glass adherence, good homogeneity, and cheap desolvation through solvent exchange. Moreover, dip coating represents a facile, affordable, and convenient technique that offers control over the angle and speed of the substrate immersion and removal from the liquid of interest [42,43,44,45]. This method has been selected for gel deposition as it has been previously effectively used by our research group for the obtaining of various organic thin films embedded with magnetic nanoparticles [45,46,47].

**Figure 1:**
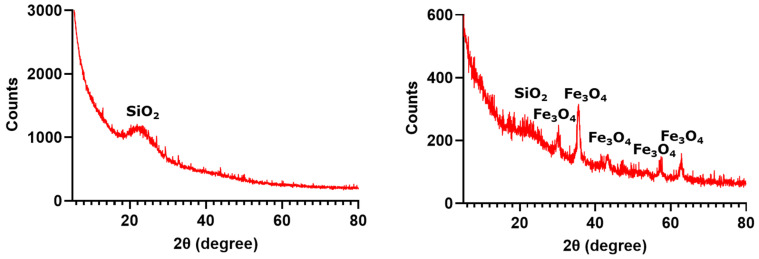
X-ray diffractograms for silica aerogel reference (**left**) and silica aerogel with Fe_3_O_4_ nanoparticles (**right**).

X-ray diffractograms have been included below (Figure 1) for silica aerogels with and without Fe_3_O_4_ nanoparticles. The XRD pattern of the silica aerogel reference exhibits a prominent broad peak with the center at a Bragg diffraction angle of 22°, which is characteristic of amorphous SiO_2_. In contrast, the diffractogram for the magnetic aerogel contains the specific peaks for magnetite. Specifically, the presence of peaks at the 2θ values of 30.18°, 35.42°, 43.22°, 53.62°, 57.18°, and 62.85° is associated with the (220), (311), (400), (422), (511), and (440) planes, respectively, which, according to PDF-ICDD database, identifies the sample as single-phase magnetite with cubic spinel crystallographic structure.

**Figure 2:**
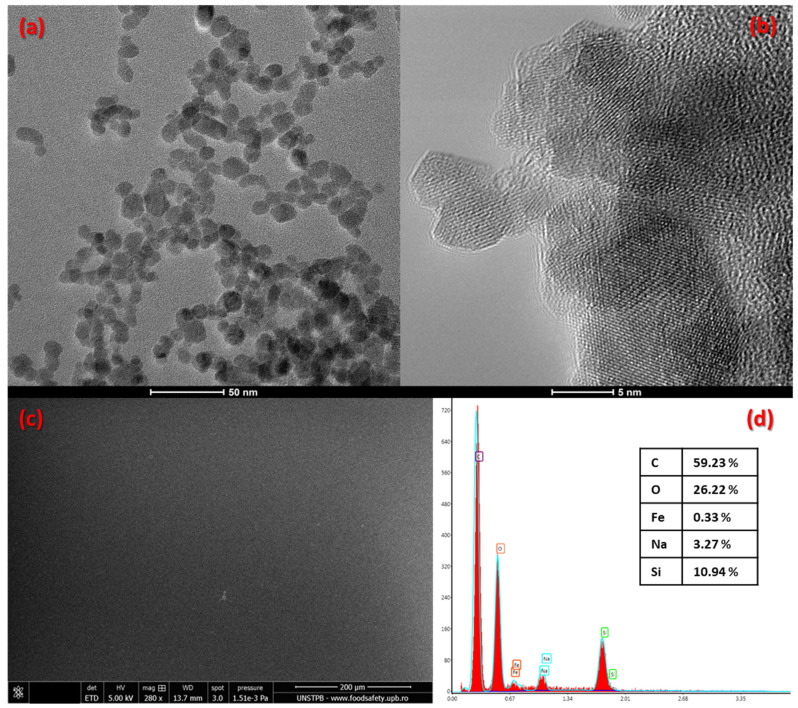
(**a**,**b**) TEM micrographs of Fe_3_O_4_ nanoparticles. (**c**) Top-view SEM micrograph and (**d**) EDS analysis for thin films of silica aerogel with Fe_3_O_4_ nanoparticles.

The core-shell nanoparticles incorporated in the magnetic aerogels were additionally characterized via transmission electron microscopy (TEM). The bright-field and high-resolution TEM micrographs (Figure 2a,b) revealed the obtaining of ultra-small nanoparticles with exclusive spherical morphology, uniform size, and reduced aggregation tendency.

The surface morphology and structure of the deposited magnetic aerogel films were analyzed with the aid of scanning electron microscopy (SEM). The typical top-view SEM image (Figure 2c) provides information on the uniformity of the coating that completely covers the glass substrate. Moreover, a uniform particle distribution was obtained within the thin film, with homogeneous spherical Fe_3_O_4_ nanoparticles being spread on the entire surface of the analyzed sample. The uniformity of the nanostructured coatings is an indicator of the well-chosen film deposition method but may also be attributed to the controlled properties of the magnetic nanoparticles. The use of spherical iron oxide-based nanoparticles with narrow size distribution was possible due to the advantages offered through their microfluidic synthesis, an emerging fabrication route recognized for its tight control over operating parameters that leads to highly stable, uniform, (near) monodispersed particles [48,49,50,51].

SEM investigations were complemented by energy-dispersive X-ray (EDS) analysis. From the EDS spectra displayed in Figure 2d, the presence of expected elements contained in the silica-based aerogel matrix and embedded iron oxide nanoparticles was confirmed. Specifically, there were identified peaks that correspond to carbon (from the nanoparticles’ shell and alginate matrix), oxygen and iron from magnetite core-shell nanoparticles, and sodium and silicon from the aerogel matrix.

**Figure 3:**
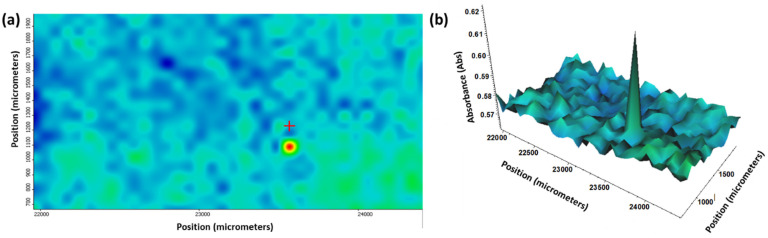
(**a**) 2D and (**b**) 3D representations of IR mappings of silica aerogel films.

**Figure 4:**
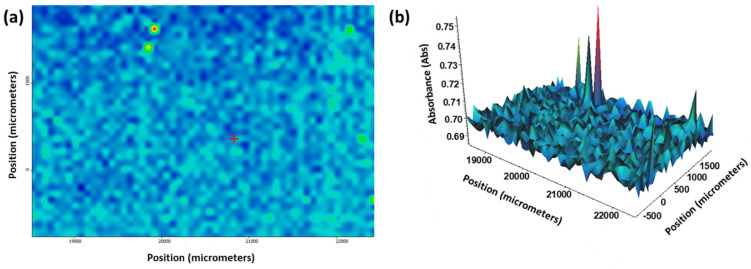
(**a**) 2D and (**b**) 3D representations of IR mappings of silica aerogel films with Fe_3_O_4_ nanoparticles.

The homogeneity of the fabricated aerogel-based thin films could also be observed through Fourier transform infrared (FT-IR) microscopy. Absorbance maps obtained for both plain silica aerogel films (Figure 3) and the thin films incorporating Fe_3_O_4_ nanoparticles (Figure 4) demonstrated the efficiency of dip coating for thin film deposition.

Further, the thin film pesticide extraction capacity from the water was successfully tested via high-resolution mass spectrometry 15 T Fourier transform ion cyclotron resonance (HR-MS FT-ICR) analysis using ultrapure water samples spiked with pesticides at ppb levels. Recent literature studies have alternatively focused on using other types of magnetic aerogel structures to remove various pollutants. Specifically, carbon-based magnetic aerogels were utilized for adsorbing organic dyes [52,53,54], organic solvents and oils [55,56], and heavy metals [54,57]. To our knowledge, only one other study has priorly employed the use of Fe–silica aerogel composite, which tested the ability of the material to adsorb malachite green [58]. However, this previous study assumed the use of aerogel as bulk instead of thin films.

**Figure 5:**
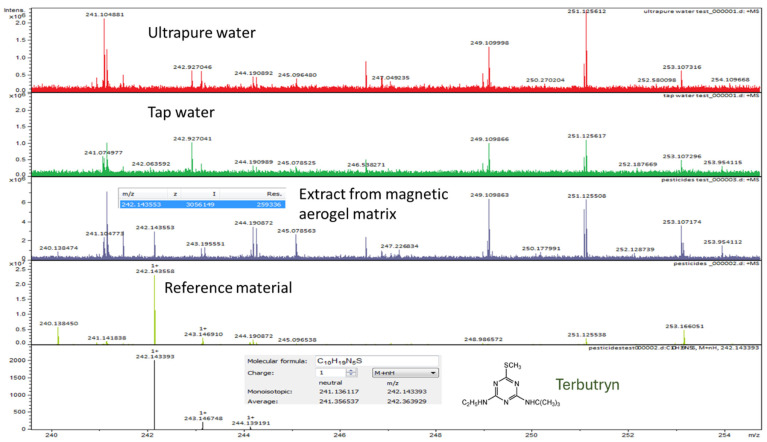
HR-MS FT-ICR—example of pesticide identification.

The qualitative evaluation revealed the existence of pesticides within the silica-based aerogel samples, proving their ability to adsorb the organic pollutants from contaminated water and highlighting their potential for future environmental remediation and solid-phase extraction (sample prep for further analysis) applications. Figure 5 displays the comparison of several spectra obtained through the HR-MS FT-ICR system. Comparing the spectra of the solution extracted from the aerogel matrix after water decontamination and contaminant reference, it could be identified with high precision that the peaks characteristic to the pesticide are present in the spectrum of the extract, and also corresponding to the simulated spectra of terbutryn. Moreover, the presence of other peaks from the extract was noticed to be attributed to potential impurities from ultrapure water. Therefore, this analysis allowed the qualitative demonstration of pesticide removal from the contaminated water sample.

**Figure 6:**
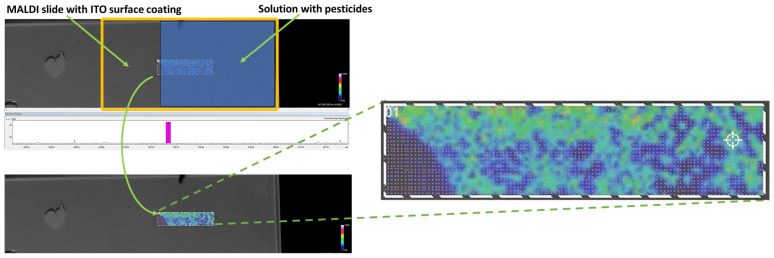
MALDI HR-MS FT-ICR analysis images displaying pesticide (i.e., terbutryn) distribution in silica aerogel with Fe_3_O_4_ nanoparticles.

Additional investigations were performed using the matrix-assisted laser desorption/ionization (MALDI) system of the HR-MS FT-ICR equipment. The obtained MALDI images (Figure 6) evidenced good thin film homogeneity and efficient pesticide adsorption within the aerogel matrix.

To our knowledge, this is the first time the HR-MS FT-ICR imaging technique was utilized to characterize such aerogel films and their efficacy for water decontamination. Up to now, HR-MS FT-ICR and other spectrometer-coupled microscopy techniques have been mostly employed in biomedical research, especially for analyzing complex biological samples (e.g., distribution of analytes in tumor models [59], localization of receptors in cancer cell lines [60], lipid imaging [61], and metal distribution [62] in brain tissues).

Nonetheless, the HR-MS FT-ICR imaging technique represents a valuable method for characterizing aerogel-based materials. Silica aerogel-based structures are particularly fitted for this analysis, given their stability and well-understood chemistry. Thus, applying this technique for the designed thin films allows a better focus on the surface chemistry and adsorbed substances within the pores of aerogels rather than on the material itself. Other aerogel-based materials (e.g., carbon aerogels [63] and aerogels doped with various metals or modified with functional groups [64]) may be subjected to similar characterization methods, while organic aerogels may have too fragile structures for HR-MS FT-ICR imaging. Therefore, even though HR-MS FT-ICR imaging can be applied to a wide range of aerogel-based materials, the specific features of the material and the intended analysis reflect its effectiveness and dictate the exact approach and any necessary modifications to the technique.

Overall, the utilized advanced imaging techniques have significant initial and operational costs. However, this characterization approach brings benefits in terms of precision, efficiency, and long-term savings that justify the investment. Addressing accessibility difficulties through cost-cutting measures, collaborative efforts, and securing funding can enhance their economic viability, allowing these imaging techniques to become integral to developing efficient and sustainable water purification technologies. Moreover, the increased demand for effective and sustainable water purification solutions may increase the economic feasibility of these systems. As market demand rises, economies of scale can lower costs and boost the accessibility of proposed investigation methods.

Additionally, the use of advanced characterization techniques allows the optimization of aerogel-based thin films toward obtaining more performant adsorbent materials. Such gel structures can be employed for the removal of organic contaminants (e.g., organic dyes, pesticides, polycyclic aromatic hydrocarbons, organic solvents, and oils) and different heavy metal ions (e.g., Cr(III), Cr(IV), As(V), Cd(II), Cu(II), and Pb(II)) [65,66,67]. Moreover, interesting studies have revealed the potential of aerogels for gas adsorption, opening the door for air purification [68,69,70] and CO_2_ conversion applications [71,72,73]. Thus, the fabricated materials deserve further investigations to assess their potential for additional uses along with pesticide adsorption.

## 3. Conclusions and Future Perspectives

The present work focuses on advanced imagining characterization methods of aerogel thin films obtained through the dip-coating deposition method with/without the addition of core-shell nanoparticles. The purpose of the magnetic behavior of the obtained thin films is related to further developments involving electromagnetic-assisted improved desorption of the adsorbed compounds. The main achievements can be summarized as follows: (i) a new microfluidic route for high-yield magnetic core-shell nanoparticle synthesis (assuring near monodisperse nanoparticles), using novel vortex type mixing chamber design of the microfluidic reaction device; (ii) advanced imaging experimental model for film homogeneity and adsorption characteristics highlighted via FT-IR microscopy and MALDI-HR-MS; (iii) confirmation of film homogeneity and surface morphology through SEM and TEM analyses; (iv) qualitative methodology for pesticide adsorption capacity of the aerogel based thin films based on HR-MS data.

This study also presents some limitations, especially as it is primarily focused on pesticide adsorption. Additional investigations should be centered on other common pollutants, such as heavy metals, pharmaceuticals, and organic dyes, to verify the material’s potential for the simultaneous extraction of several pollutant species. To broaden the applicability of the designed aerogel-based films, their efficiency should be tested for decontamination of real water samples from contaminated water bodies (instead of artificially contaminated solutions).

Moreover, scaling up the fabrication may also pose challenges, given that, despite its advantages, the dip coating method has not been explored at the industrial level. Therefore, future perspectives should also be directed toward optimizing the synthesis process for large-scale production. In addition, when moving to large-scale utilization, future studies would be directed to other aspects as well, including long-term performance, durability, and reusability of the aerogel films in real-world conditions and analyses on the environmental impact of the synthesis, usage, and disposal of proposed materials.

Thus, the results presented in this work aim to serve as an inception point for further research, encouraging more in-depth investigations. The obtained materials, methodologies, and analytical protocols will be able to sustain further developments related to aerogel composite applications for water decontamination purposes and novel solid-phase extraction sample preparation for fast water body screenings based on thin film microfluidic devices.

## 4. Materials and Methods

### 4.1. Materials

For nanoparticle preparation, the following were used: iron oxide precursors (i.e., ferric chloride—FeCl_3_ and iron sulfate heptahydrate—FeSO_4_·7H_2_O) purchased from Sigma Aldrich Merck (Darmstadt, Germany), salicylic acid bought from ATOCHIM PROD (Bucharest, Romania), sodium hydroxide (NaOH) acquired from Lach-Ner (Tovarni, Czech Republic), and acetic acid purchased from Emsure Merck Millipore (Darmstadt, Germany).

Additional reagents were employed to form aerogel thin films. Specifically, alginic acid, cetyltrimethylammonium bromide, ammonium bicarbonate, sodium trisilicate, and calcium chloride purchased from Sigma Aldrich Merck were supplementarily used.

Being of analytical purity, all reagents were used as received. Ultrapure water was used for all experiments.

### 4.2. Aerogel-Based Thin Film Preparation

**Figure 7:**
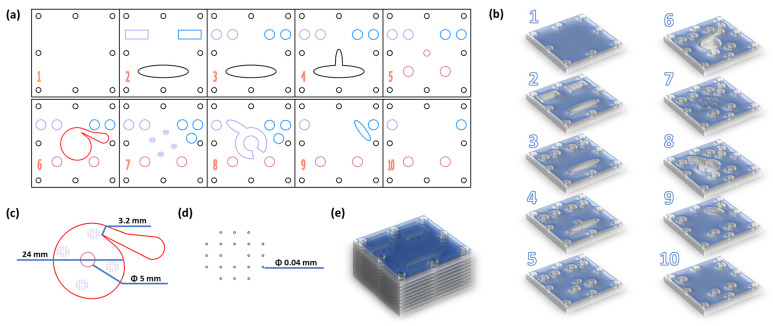
Microfluidic platform configuration. (**a**) Schematic 2D and (**b**) 3D representations of the microfluidic platform individual layers. (**c**) Overlayed reaction area and its dimensions; (**d**) Reactant inlet dimensions (from layer 7). (**e**) 3D representation of the microfluidic assembly (overlapped layers). Colors: purple—solution 1 inlet channels, blue—solution 2 inlet channels, red—vortex mixing chamber, pink—outlet channels.

**Figure 8:**
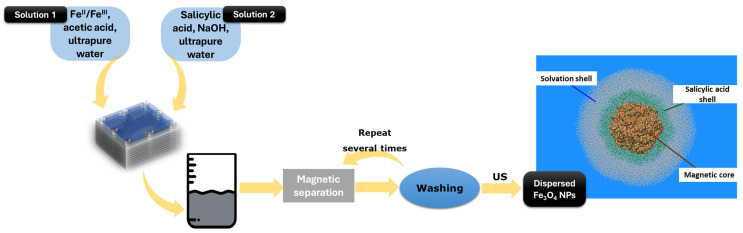
Flowchart for the synthesis of magnetic core-shell nanoparticles.

Core-shell iron oxide nanoparticles were synthesized using a custom-built multilayered microfluidic platform supposing a vortex mixing chamber (Figure 7). Similar to our prior works on microfluidic syntheses [49,74,75], two solutions were introduced in the inlets of the microfluidic chamber with the aid of a peristaltic pump, and products were collected, magnetically separated, washed, and further ultrasonically dispersed in ultrapure water. In more detail, Solution 1 contained iron oxide precursors (stoichiometric ratio of iron II/III ions) dissolved in ultrapure water, while Solution 2 contained sodium hydroxide and salicylic acid dissolved in an equal volume of ultrapure water. For clarity, the synthetic process has been schematically represented in Figure 8.

**Figure 9:**
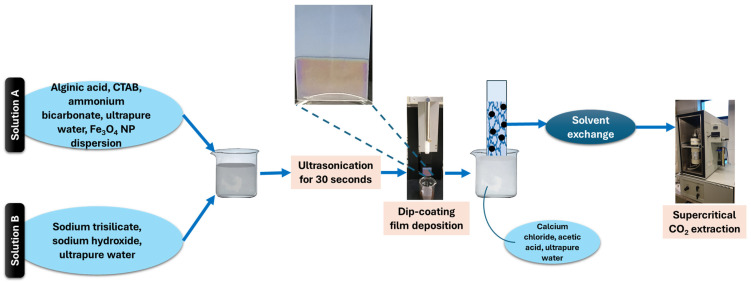
Flowchart for aerogel-based thin film fabrication.

The obtained magnetic nanoparticle dispersion was used alongside alginic acid, cetyltrimethylammonium bromide, ammonium bicarbonate, and ultrapure water to create one of the solutions (Solution A) required for aerogel formation. The second solution (Solution B) consisted of a mixture of sodium trisilicate, sodium hydroxide, and ultrapure water. Solutions A and B were mixed together and homogenized with the aid of an ultrasonic processor for 30 s (Figure 9). Several optimization stages were performed in order to ensure a significantly increased gelation time correlated with the requirements of the dip-coating deposition method.

Further, a glass slide was introduced in the mixture using a dip-coating device at a constant immersion speed of 200 mm/min. After thin film deposition, the dip-coating device slowly removed (30 mm/min) the glass slide from the solution. The slide was further immersed in a solution of acetic acid and calcium chloride to ensure gel formation. Then, the film was washed several times with water and ethanol. To create the characteristic aerogel structure, the thin gel films were dried via supercritical CO_2_ extraction (5 h—static, 3 h—dynamic operation mode).

As a reference material, aerogel thin films without magnetic nanoparticles were also fabricated in a manner similar to the one described above.

### 4.3. Transmission Electron Microscopy (TEM)

For TEM analysis, the nanoparticle sample was first dispersed in ethanol via ultrasonic treatment for 15 min; then, it was placed onto a 400-mesh lacey carbon-coated copper grid and dried at room temperature. Micrographs were captured with the aid of a ThermoFisher Scientific 80–200 Titan Themis transmission electron microscope (Hillsboro, OR, USA). Data collection was performed utilizing the transmission mode of the equipment operated at 200 kV, with point and line resolutions of 2 Å and 1 Å, respectively.

### 4.4. Scanning Electron Microscopy (SEM)

The morphological and dimensional characteristics of the thin films were evaluated using a Versa 3D scanning electron microscope model coupled with an energy-dispersive spectrometer (EDS) from Thermo Fisher—FEI (Eindhoven, The Netherlands). Using a carbon-bearing strip, the samples were fixed and inserted into the analysis chamber of the microscope. The micrographs were acquired by achieving the resulting secondary electron beam with an energy of 10 keV.

### 4.5. Fourier Transform Infrared (FT-IR) Microscopy

For determining the homogeneity of thin film deposition, 2D and 3D maps of the films were acquired using a Thermo Nicolet iN10 MX FT-IR microscope from Thermo Fischer Scientific (Waltham, MA, USA), equipped with a cooled detector. The scans were collected in reflection mode, 4000–650 cm^−1^ spectral range, 5 scan accumulation, resolution 8 cm^−1^.

### 4.6. X-ray Diffraction (XRD)

The crystallinity of the samples was evaluated using a PANalytical Empyrean (PANalytical, Almelo, The Netherlands) model diffractometer supplied with a hybrid monochromator (2xGe 220) on the incident side and a parallel plate collimator mounted on PIXcel 3D detector on the diffracted side. The measurements were performed at room temperature by means of grazing incidence X-ray diffraction (GIXRD), with the following parameters: angle of incidence ω = 0.5° for Bragg angle values of 2θ at intervals of 5° and 80°. The diffractometer radiation is Cu Kα with λ = 1.5406 Å (40 mA and 45 kV).

### 4.7. Matrix-Assisted Laser Desorption/Ionization—High-Resolution Mass Spectrometry 15 T Fourier Transform Ion Cyclotron Resonance (MALDI-FT-ICR-MS)

MALDI slides (conductive ITO-coated glass slides) were used as the substrate for the described aerogel film depositions and further immersed in pesticide-spiked samples, respectively, tap water. After desiccation and sinapinic MALDI matrix application, FT-ICR-HR-MS-MALDI analysis was performed in order to prove the pesticide adsorption on the thin film aerogel matrix (using the MALDI sample holder of the Bruker Solarix 15T HR-MS-FT-ICR system). MALDI parameters: positive ionization, minimum laser focus at 30% of laser power, 500 laser pulses, 1500 Hz laser frequency, and a scanning resolution of 100 microns were employed.

Pesticide-spiked samples were prepared by dissolving the desired amount of pesticide (e.g., terbutryn) in ultrapure water to achieve the required concentration (e.g., ppb levels). Further, the aerogel-coated slides were immersed in the pesticide-contaminated water sample and allowed to adsorb the organic pollutants for 30 min.

## Data Availability

The data presented in this study are available on request from the authors.
